# Supplementary material for: Sperm motility in mice with oligo-astheno-teratozoospermia restored by in vivo injection and electroporation of naked mRNA
Source: eLife. 2026 Mar 3;13:RP94514. doi: 10.7554/eLife.94514 (PMC12956281; doi:10.7554/eLife.94514)
Supplement: Figure 1—figure supplement 1—source data 1. [file elife-94514-fig1-figsupp1-data1.zip › figure_1-_supplement_1-source_data_1.pdf]

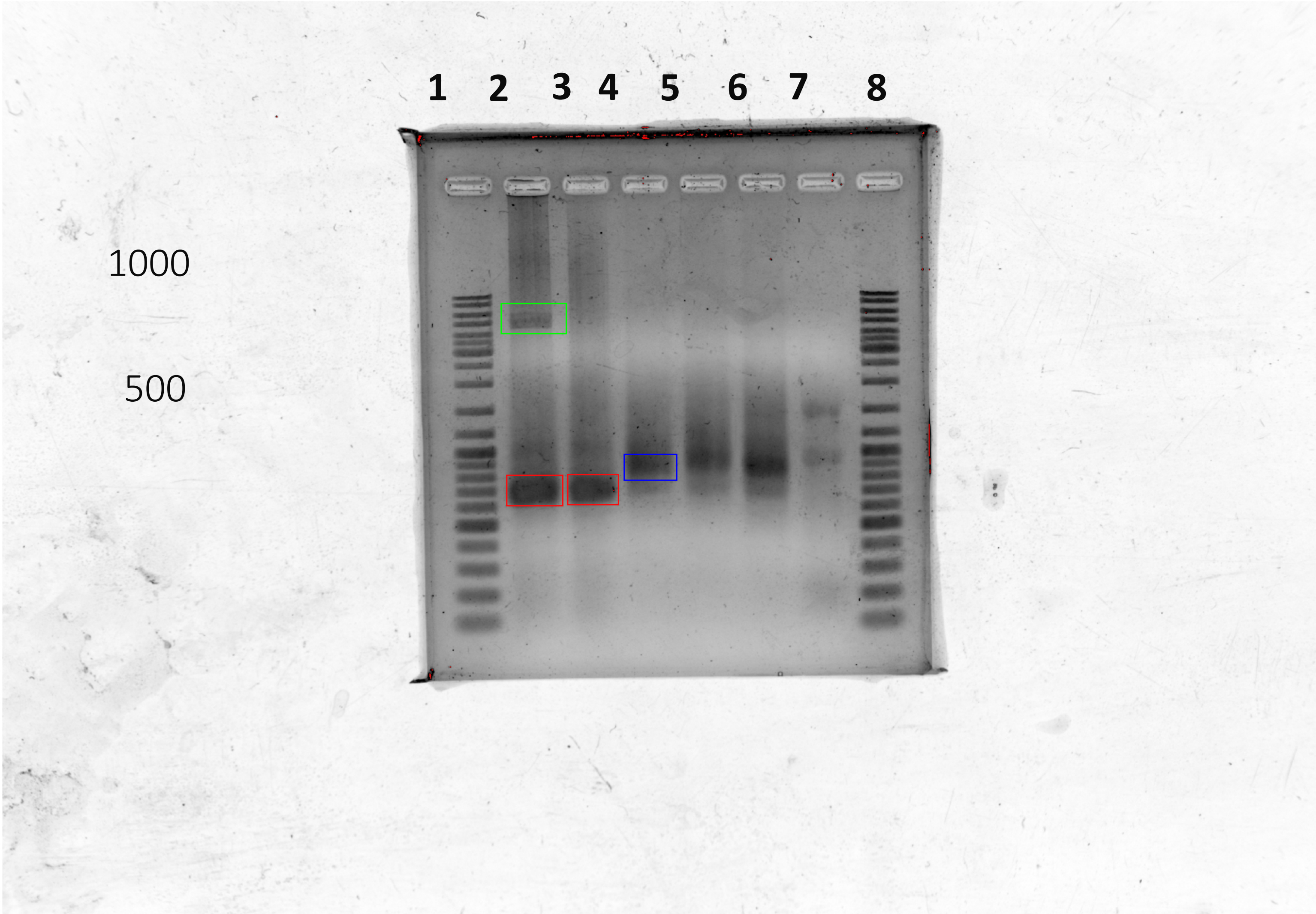

1 and 8. DNA Ladder
2.mCherry -mRNA before DNASe treatment
3. mCherry- mRNA after DNAse treatment
4. mCherry-mRNA after 30 min Poly A tailing
5. mCherry-mRNA after 60 min Poly A tailing
6. mCherry-mRNA after elution
7. mRNA standart 2 from Quibit assay

Figure 1—figure supplement 1, Source Data 1.
Original membranes corresponding to Figure 1—figure supplement 1D.
Relevant bands are highlighted by rectangles indicating mCherry plasmid (green), mCherry mRNA before tailing (red), and poly(A)-tailed mCherry mRNA (blue).
