# Supplementary figures and images for: Sperm motility in mice with oligo-astheno-teratozoospermia restored by in vivo injection and electroporation of naked mRNA
Source: eLife. 2026 Mar 3;13:RP94514. doi: 10.7554/eLife.94514 (PMC12956281; doi:10.7554/eLife.94514)

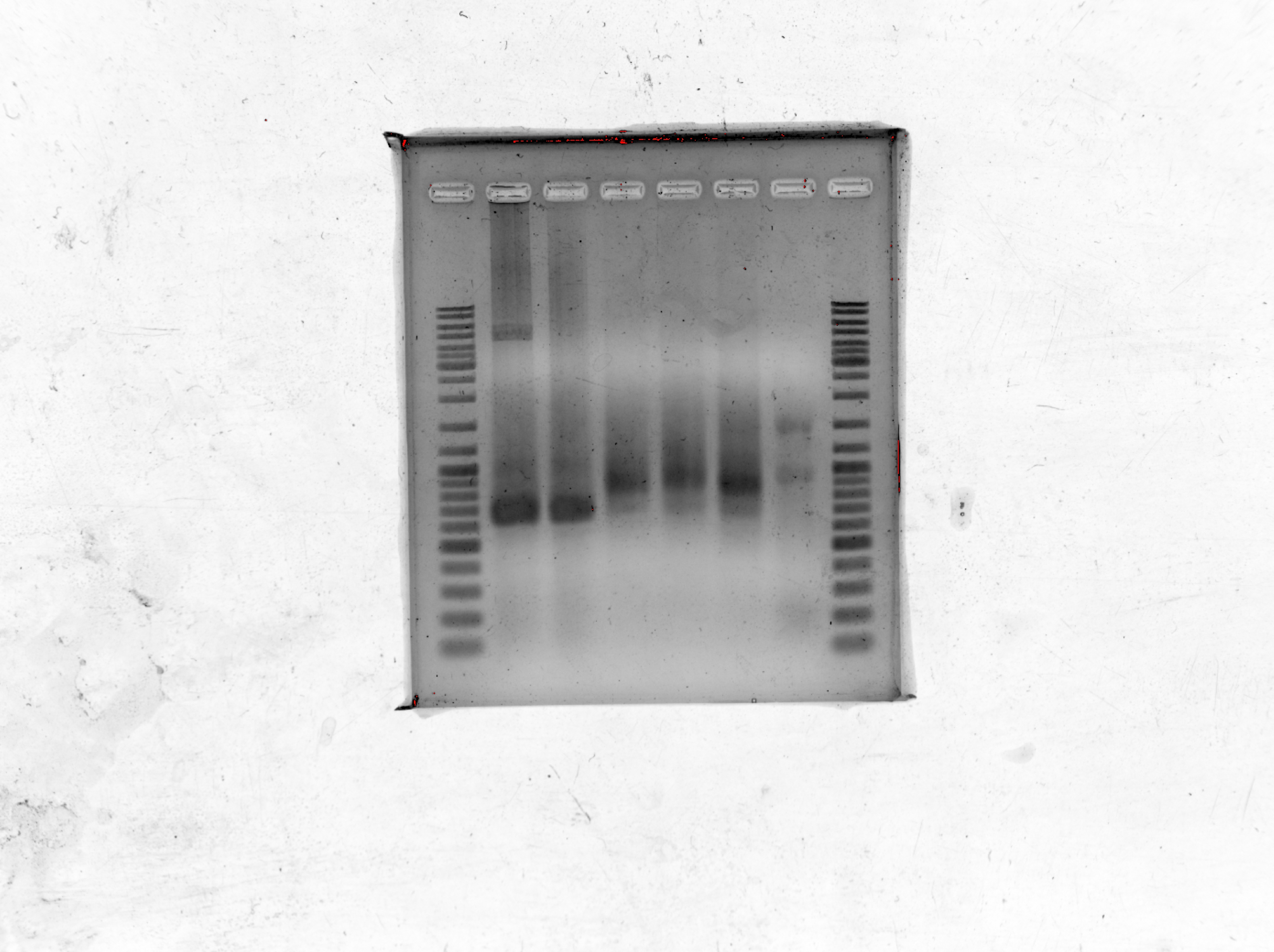

Supplement: Figure 1—figure supplement 1—source data 2. [file elife-94514-fig1-figsupp1-data2.zip › figure1-supplement 1 -source data 2/ChemiDoc XRS 2021-01-06 16hr 18min m cheery.jpg]

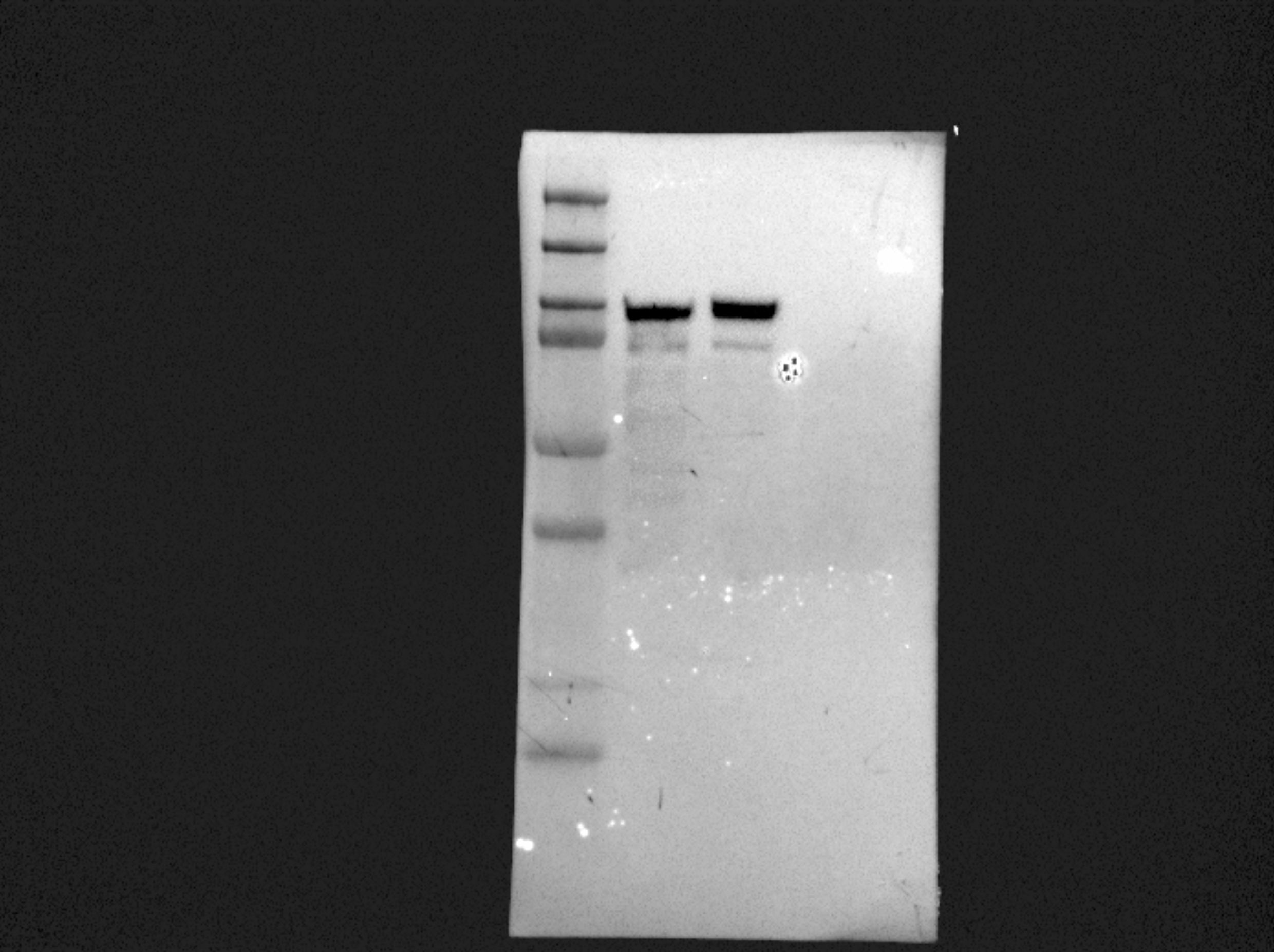

Supplement: Figure 12—figure supplement 1—source data 2. [file elife-94514-fig12-figsupp1-data2.zip › Figure12-supplement1-source data 2/fiinal2ChemiDoc XRS 2021-09-29 09hr 14min_Exposure_7.0sec+pmChemiDoc XRS 2021-09-29 09hr 12min.jpg]

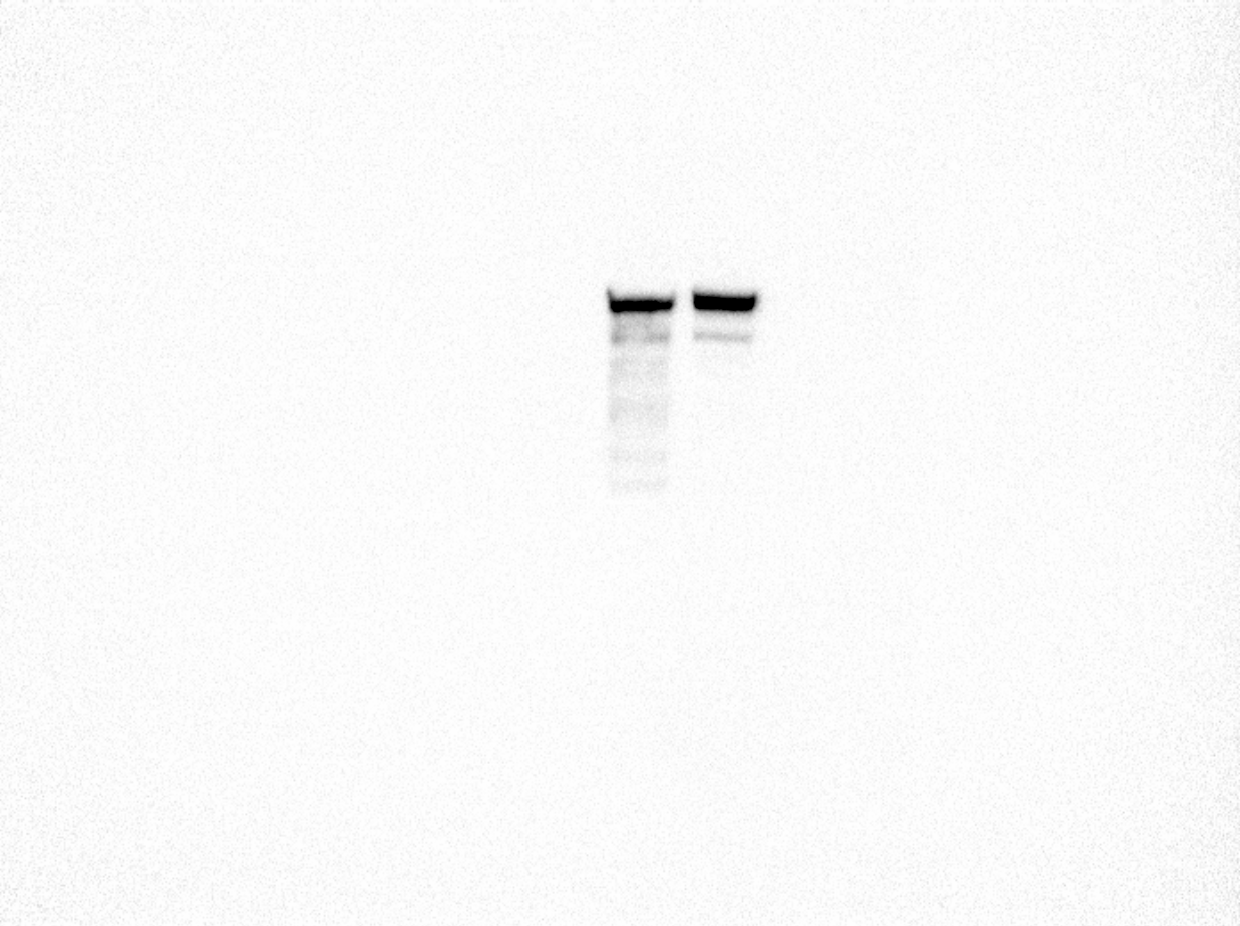

Supplement: Figure 12—figure supplement 1—source data 2. [file elife-94514-fig12-figsupp1-data2.zip › Figure12-supplement1-source data 2/fiinalChemiDoc XRS 2021-09-29 09hr 14min_Exposure_7.0sec.tif]

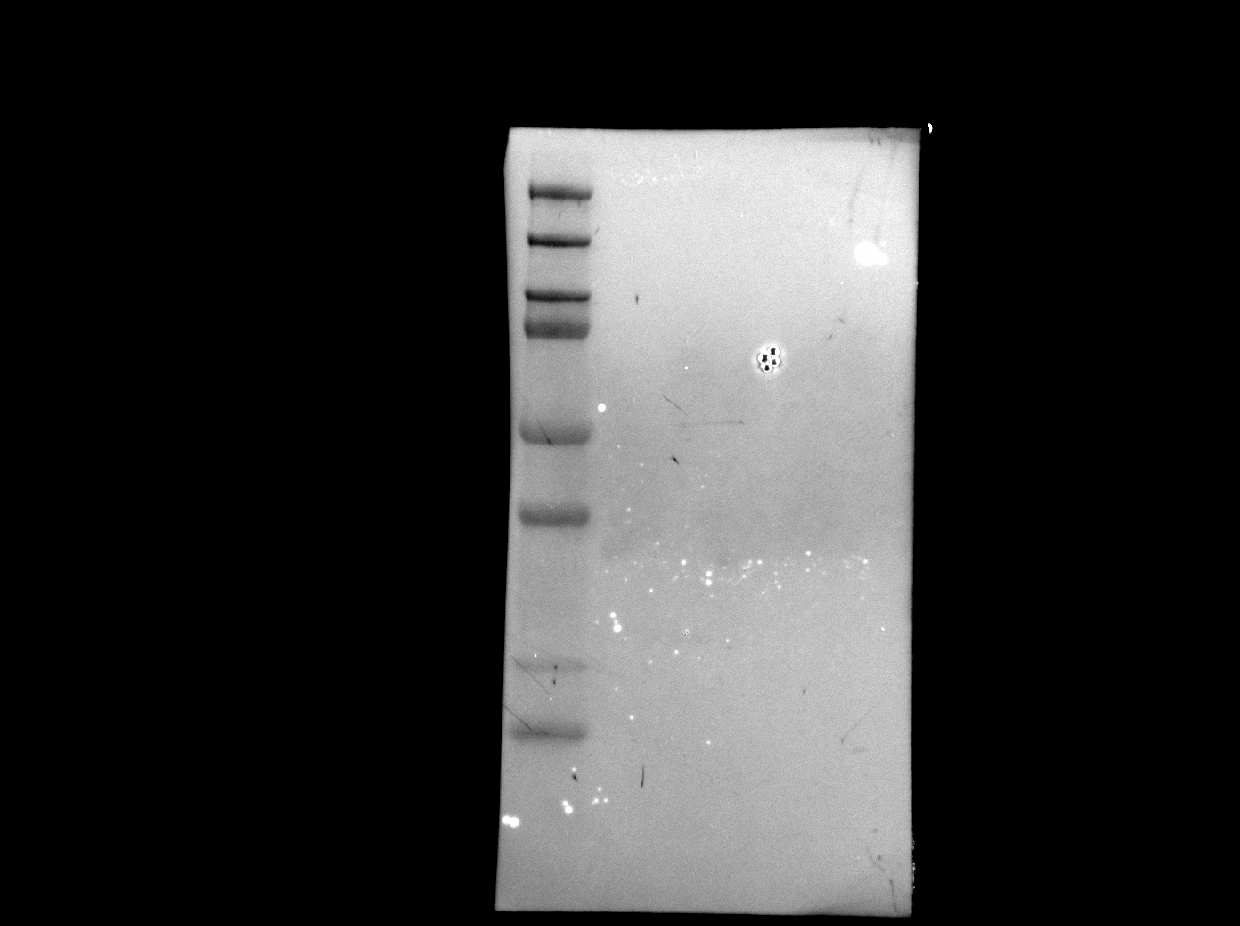

Supplement: Figure 12—figure supplement 1—source data 2. [file elife-94514-fig12-figsupp1-data2.zip › Figure12-supplement1-source data 2/pmChemiDoc XRS 2021-09-29 09hr 12min.tif]
