# Supplementary material for: Sperm motility in mice with oligo-astheno-teratozoospermia restored by in vivo injection and electroporation of naked mRNA
Source: eLife. 2026 Mar 3;13:RP94514. doi: 10.7554/eLife.94514 (PMC12956281; doi:10.7554/eLife.94514)
Supplement: Figure 12—figure supplement 1—source data 1. [file elife-94514-fig12-figsupp1-data1.zip › Figure_12XXXfigure_supplement_1-source_data_1.pdf]

Figure 12-supplement 2 , Source Data 1. Original membranes corresponding to Figure 12-supplement 2, panel D. The lane corresponds to ARMC2 protein

1.Protein Ladder

2. HEK + *Armc2*-mRNA

3.HEK + *Armc2*-EEV

4.HEK NT

1 2 3 4

KDa

250

150

100

75

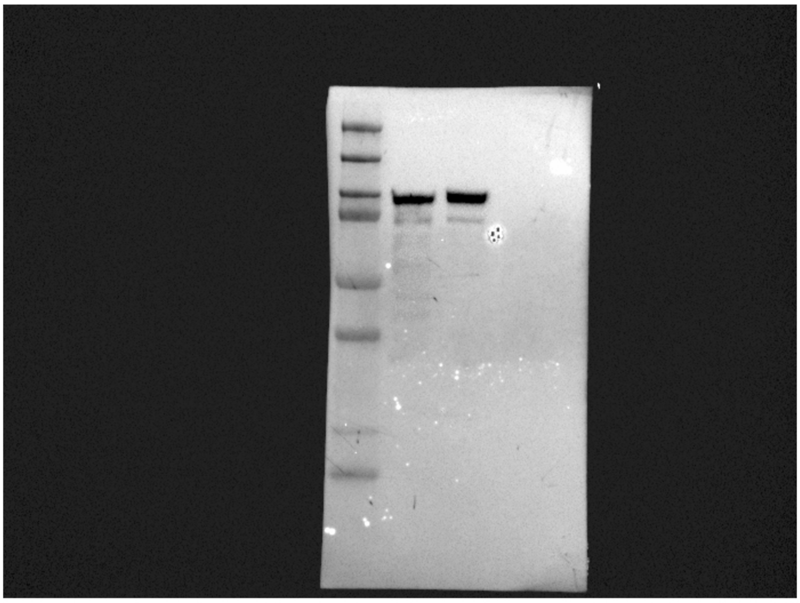

α-HA
(merged HA and PM)

1 2 3 4

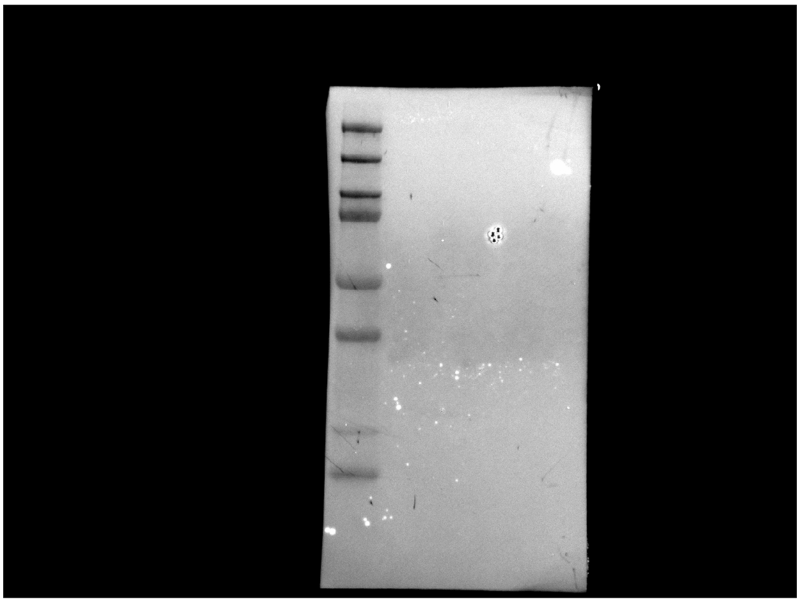

Colorimetric

1 2 3 4

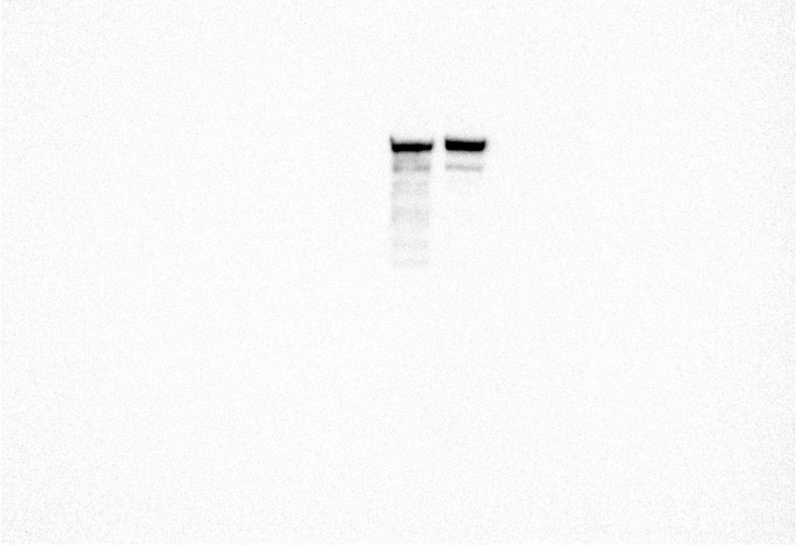

α-HA
